# A Broad Spectrum Lasso Peptide Antibiotic Targeting the Bacterial Ribosome

**DOI:** 10.21203/rs.3.rs-5058118/v1

**Published:** 2024-09-16

**Authors:** Gerard Wright, Manoj Jangra, Dmitrii Travin, Elena Aleksandrova, Manpreet Kaur, Lena Darwish, Kalinka Koteva, Dorota Klepacki, Wenliang Wang, Maya Tiffany, Akosiererem Sokaribo, Brian Coombes, Nora Vázquez-Laslop, Yury Polikanov, Alexander Mankin

**Affiliations:** McMaster University; McMaster University; University of Illinois at Chicago; University of Illinois at Chicago; McMaster University; McMaster University; McMaster University; University of Illinois at Chicago; McMaster University; McMaster University; McMaster University; McMaster University; University of Illinois at Chicago; University of Illinois at Chicago; University of Illinois

**Keywords:** Lasso peptide, RiPP, ribosome, antibiotic, tRNA, decoding, translocation, inhibitor, drug resistance

## Abstract

Lasso peptides, biologically active molecules with a distinct structurally constrained knotted fold, are natural products belonging to the class of ribosomally-synthesized and posttranslationally modified peptides (RiPPs). Lasso peptides act upon several bacterial targets, but none have been reported to inhibit the ribosome, one of the main antibiotic targets in the bacterial cell. Here, we report the identification and characterization of the lasso peptide antibiotic, lariocidin (LAR), and its internally cyclized derivative, lariocidin B (LAR-B), produced by *Paenabacillussp*. M2, with broad-spectrum activity against many bacterial pathogens. We show that lariocidins inhibit bacterial growth by binding to the ribosome and interfering with protein synthesis. Structural, genetic, and biochemical data show that lariocidins bind at a unique site in the small ribosomal subunit, where they interact with the 16S rRNA and aminoacyl-tRNA, inhibiting translocation and inducing miscoding. LAR is unaffected by common resistance mechanisms, has a low propensity for generating spontaneous resistance, shows no human cell toxicity, and has potent *in vivo* activity in a mouse model of Acinetobacter *baumannii* infection. Our finding of the first ribosome-targeting lasso peptides uncovers new routes toward discovering alternative protein synthesis inhibitors and offers a new chemical scaffold for developing much-needed antibacterial drugs.

## INTRODUCTION

Antibiotic resistance is a global crisis, responsible for more than 4.5 million deaths in 2019, threatening our ability to treat bacterial infections effectively^1^. Consequently, identifying new antibacterials is a top priority. Various microbially produced peptide-based antibiotics, ranging from glycopeptides like vancomycin, cationic peptides such as colistin, and beta-lactams such as penicillins and cephalosporins, have been successfully deployed as medicines for the treatment of diseases caused by bacterial pathogens. Most medically relevant peptide antibiotics are produced non-ribosomally via condensation of proteinogenic and non-canonical amino acids by the specialized peptide synthetase assembly lines encoded in the genomes of antibiotic-producing bacteria and fungi^2,3^.

A rapidly expanding class of antibiotics comprises ribosomally-synthesized and posttranslationally modified peptides (RiPPs)^4–6^. These bioactive natural products are generated via ribosomal translation of the peptide-encoding gene and subsequent processing of the precursor peptide by dedicated enzymes that introduce a variety of posttranslational modifications, including intramolecular cyclization, dehydration, formation of heterocycles, etc. The modifications set the three-dimensional (3D) shape of the peptides, facilitate interaction with the target, and protect them from degradation by cellular peptidases. Among RiPPs, a special place belongs to lasso peptides (LPs). LPs have a unique 3D shape with their C-terminal tail threaded through an intramolecular ring formed via an isopeptide bond between the N-terminal backbone amine and the side chain carboxyl of an internal Asp or Glu residue^7–9^. The result is a highly stable spatially knotted lariat structure that gives the class its name. Several of the discovered LPs have antibacterial properties. For example, lassomycin targets microbial ClpP protease^10^ and exhibits antimycobacterial activity, whereas microcin J25 and capistruin, active against Gram-negative bacteria, block the action of RNA polymerase^11,12^. However, no LPs have been shown to act upon the ribosome.

The ribosome, responsible for genetically programmed protein synthesis, is a privileged antibiotic target. A large variety of chemically distinct molecules stop bacterial growth by targeting their ribosomes and interfering with protein synthesis^13,14^. Most known antibiotics bind at a few well-characterized functional sites, such as the decoding center in the small ribosomal subunit, the catalytic peptidyl transferase center, or the nascent peptide exit tunnel in the large subunit. However, increasingly common resistance genes encoding rRNA-modifying or drug-modifying enzymes can render bacteria immune to many inhibitors acting upon the ‘overused’ antibiotic sites, often providing cross-resistance to chemically distinct antibiotic classes^15–17^. Therefore, finding ribosomal inhibitors acting upon distinct sites, exhibiting novel modes of action, and having unconventional structures offers attractive opportunities for developing new medicines.

Here, we report the discovery of the first ribosome-targeting LP, lariocidin (LAR), that binds to a new ribosomal site, inhibits translation elongation, and induces miscoding. We show that LAR possesses broad antimicrobial activity inhibiting the growth of pathogenic Gram-positive and Gram-negative bacteria and is active in a bacterial infection animal model.

## RESULTS

### The discovery of lariocidins, novel LPs with antibacterial properties.

In our quest for new antibiotics, we generated a collection of environmental bacterial strains by growing them on soil-agar plates at room temperature for approximately one year. Such an extended incubation period aimed to reveal even the slowest, often overlooked bacteria to grow. After regrowing individual colonies in rich medium, their methanolic extracts were partially fractionated by reversed-phase chromatography (RPC)^18^ and tested against a clinical multi-drug resistant (MDR) isolate of the Gram-negative pathogen *Acinetobacter baumannii* and the antibiotic-hypersusceptible *Escherichia coli* BW25113Δ*tolC*Δ*bamB*. The methanolic extract of the strain M2, identified as a Paenibacillus sp., showed potent antibacterial activity. The complete genome sequence of Paenibacillus sp. M2 revealed the presence of a biosynthetic gene cluster (BGC) of the known antibiotic colistin, which was identified in one of the RPC fractions. However, the pre-fractionated extract remained active even against a colistin-resistant *E. coli* strain (Extended Data Fig. 1a). Subsequent bioactivity-guided purification yielded a compound with a molecular mass of 1870.06 Da associated with antibiotic activity. The determined mass was distinct from those of colistins (MW 1154.7 and 1168.7 Da for colistin A and B, respectively) and had no matches in antibiotic databases (**Extended Data Fig. 1b, c**).

**Figure 1:**
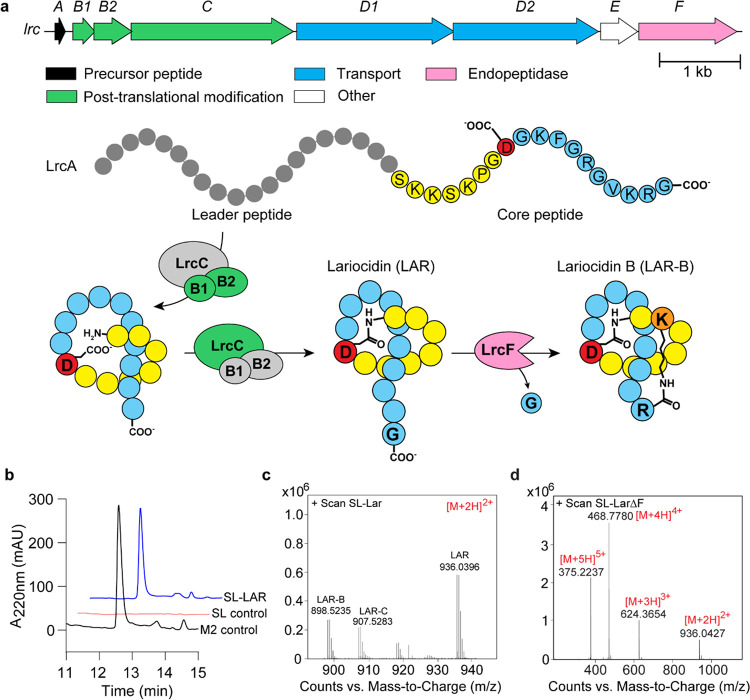
Lariocidin and its biosynthetic gene cluster. (**a**) Top, gene composition of the *lrc* BGC. Bottom, posttranslational modification of the LrcA precursor peptide leads to the production of LAR-A and LAR-B. (**b**) Heterologous expression of *lrc* BGC in *Streptomyces lividans* and analysis of LAR in cell-free supernatant. The panel shows chromatographic analysis of LAR produced in the heterologous host. SL-Lar S. lividans pIJ10257-*lrc*; M2 control is LAR purified from the native producer *Paenibacillus* M2; SL-control is *S. lividans* with the empty vector (without lrc BGC). (**c**) LC-MS analysis of LAR purified from the heterologous host. All masses shown are ions corresponding to [M+2H]^2+^. (**d**) LC-MS analysis of LAR produced by heterologous host from the *lrc* operon with the *lrcF* gene deleted, showing exclusive production of LAR (or LAR-A), but not LAR-B and LAR-C variants.

Optimization of fermentation conditions allowed for the purification of the active compound in amounts sufficient for chemical characterization as well as biochemical and microbiological testing. Analysis of the chemical properties of the active compound pointed to the peptidic nature of the antibiotic (**Supplementary Fig. 1–9**). Further examination of the *Paenibacillus* sp. M2 genome revealed the presence of a BGC of a putative Class-II LP^7^ whose predicted molecular mass and amino acid composition matched those of the isolated active compound (Fig. 1a). Therefore, we surmised that the new antibiotic produced by *Paenibacillus* sp. M2 is an LP, which we termed lariocidin (LAR) to reflect its putative lariat structure. Refactoring and cloning of the LAR BGC into a heterologous host, *Streptomyces lividans*, resulted in the production of a compound with a molecular mass and antibiotic activity matching those of the LP isolated from *Paenibacillus sp*. M2 (Fig. 1b, c), confirming that LAR is indeed the product of expression of the identified BGC, which we named *lrc*.

Our ability to produce LAR in a heterologous host argues that the cloned *lrc* BGC contains all the genes necessary for the synthesis and secretion of the antibiotic and self-resistance. Consistent with the composition of known antimicrobial LP gene clusters (Fig. 1a), the biosynthesis of LAR involves the ribosomal synthesis of the precursor peptide LrcA, which is processed by the peptidase/lasso cyclase complex (LrcB1B2C), yielding the mature LP. The ABC-type transporter LrcD1D2 is likely responsible for the secretion of LAR from the cell of the producing bacteria. The *lrcE* gene is predicted to encode a GCN5-related N-acetyl transferase (GNAT), which we hypothesize confers self-resistance. The *lrcF* gene encodes a clostripain-family peptidase involved in the formation of LAR isoforms (see below). NMR analyses showed that mature LAR is comprised of 18 amino acids, with the first eight residues arranged into a macrolactam ring via the formation of an isopeptide bond between the amino group of the N-terminal Ser1 residue and the side chain of Asp8 with the remaining C-terminal ‘tail’ threaded through this ring; a structure later confirmed by X-ray analysis (see below) (Fig 1a; Fig. 3).

Two additional compounds termed lariocidin B and C (LAR-B and LAR-C, respectively), closely related to LAR (or LAR-A, to differentiate from other variants), were secreted by both *Paenibacillus* sp. M2 and the S. *lividans* heterologous host expressing the lrc BGC (Fig. 1; **Extended Data Fig. 1**). Mass spectrometry and X-ray crystallographic data (see below) show that LAR-B lacks the C-terminal Gly18 and features an unprecedented second intramolecular ring formed through an additional isopeptide bond between the carboxylic group of the C-terminal Arg17 and the side chain of Lys2 (Fig. 3b). Similar to Lar-B, LAR-C also lacks the Gly18, but does not have the second intramolecular ring and its C-terminal Arg17 remains unaltered. Deletion of *lrcF* from the *lrc* BGC expressed in *S. lividans* resulted in the exclusive production of LAR-A with no detectable LAR-B or LAR-C (Fig. 1d), confirming the role of the clostripain-like LrcFin cleaving of the C-terminal Gly residue of LAR. LAR-B showed antibiotic activity comparable to that of the main LAR product (**Supplementary Fig. 11**); the activity of LAR-C was not assessed due to the difficulty in isolating it in sufficient quantities. The LAR complex eluted as a single peak by HPLC (**Supplementary Fig. 1a**) and contained all three isoforms, i.e., LAR-A, LAR-B, and LAR-C, which could be separated following treatment with di-tert-butyl decarbonate (Boc anhydride). Different fermentation batches produced varying ratios of three compounds; however, LAR-A was consistently the primary product.

### Lariocidin inhibits bacterial protein synthesis.

**Figure 2:**
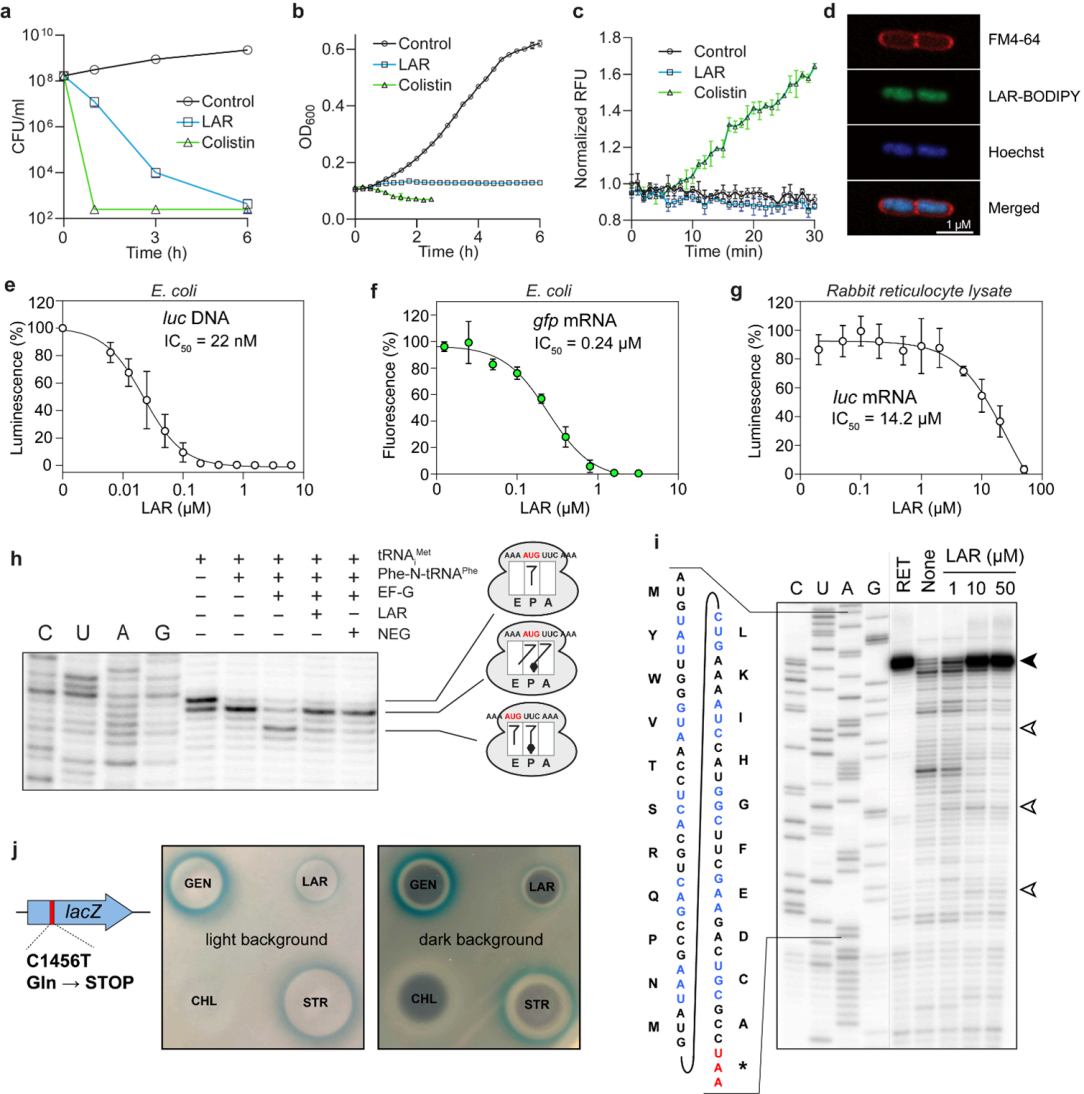
LAR exhibits bactericidal activity and targets bacterial protein synthesis. (**a**) Reduction in viable cell counts (colony forming units, CFU) of *E. coli* cultures treated with LAR at 10xMIC (40 μg/ml) or the cell membrane-targeting lytic antibiotic colistin at 10xMIC (5 μg/ml). (**b**) Effect of LAR or colistin on the density of exponential *E. coli* cell cultures. In **a** and **b**, data are presented as mean ±SD (standard deviation) of three technical replicates and are representatives of two biological replicates with similar results. (**c**) A propidium iodide accumulation assay was used to assess the effect of LAR on cell permeabilization. Colistin was used as a positive control. The y-axis represents relative fluorescence units (RFU) normalized by the initial fluorescence at time 0. Plot points represent the mean of three biological replicates, with error bars representing SD. (**d**) LAR-BODIPY accumulates in the cytoplasm of *E. coli* cells. Green; LAR-BODIPY fluorescence; red: membrane stain Fm-4–64; blue: DNA stain Hoechst 33342. (**e, f**) Effect of LAR on *E. coli* protein synthesis in the cell-free transcription-translation system programmed with firefly luciferase-encoding plasmid (**e**) or GFP mRNA (**f**). (**g**) Inhibition of protein biosynthesis in rabbit reticulocyte lysate programmed with *luc* mRNA by LAR in varying concentrations. In **e–g**, data points represent the mean of three experiments with error bars indicating SD. (**h**) LAR inhibits ribosome translocation. The pretranslocation complex is assembled from the *E. coli* ribosome, short mRNA with the AUG and UUC codons, deacylated tRNA_i_^Met^ in the P-site, and N-acetyl-Phe-tRNA^Phe^ in the A-site. The addition of elongation factor G (EF-G) and GTP promotes translocation, which is detected by extension of the primer annealed to the mRNA 3’ end. Adding LAR or a control antibiotic negamycin (NEG), a known inhibitor of translocation^62^, interferes with the movement of mRNA/tRNA complex through the ribosome. (**i**) Toeprinting analysis of ribosomes stalled on a model mRNA template in the presence of increasing concentrations of LAR. The inhibitor of translation initiation retapamulin (RET) was used as a control. ‘None’ – no antibiotic control. The bands representing ribosomes stalled at the start codon are indicated by a black arrowhead, and those of ribosomes stalled at internal mRNA codons are indicated by open arrowheads. (**j**) LAR induces miscoding as evidenced by the ability of *E. coli* cells harboring the *lacZ* gene with a premature stop-codon to produce functional β-galactosidase when exposed to subinhibitory concentrations of LAR (visualized as a blue halo around the zone of cell growth inhibition on indicator plates). The known miscoding-inducing antibiotics gentamicin (GEN) and streptomycin (STR) were used as positive controls; chloramphenicol (CHL) served as a negative control. The plate was imaged with light and dark backgrounds to reveal the blue halo reflecting β-galactosidase expression (left image) and zones of inhibition of cell growth around the drops of applied antibiotics (right image).

LAR exhibits bactericidal activity (Fig. 2a; **Extended Data Fig. 8a**). Given the density of positively charged residues in LAR, we questioned whether LAR would disrupt the cell membrane and kill bacteria by causing their lysis, similar to other cationic antimicrobial peptides, including colistin^19,20^. However, we found that while the addition of LAR to an *E. coli* culture dramatically reduced the viable cell count, it did not cause a decrease in the optical density of the culture below the initial point, which would indicate cell lysis (Fig. 2a, b). Further experiments showed that LAR did not permeabilize or depolarize the bacterial membrane (Fig. 2c; **Extended Data Fig. 2a, b**) nor altered cell morphology (**Extended Data Fig. 2c**), pointing to a non-lytic mechanism of action. Therefore, we speculated that LAR might be acting upon an intracellular target. Consistent with this hypothesis, fluorescently labeled LAR, which retained antibiotic activity (**Extended Data Fig. 3a, b**), accumulated in the cytoplasm of *E. coli* cells (Fig. 2d; **Extended Data Fig. 3c, d**).

In an attempt to reveal the putative intracellular target of LAR, we selected spontaneous resistant mutants by plating *E. coli* and *B. subtilis* cells on a solid medium supplemented with LAR. The resistant mutants appeared with a frequency of ~10^−8^. Whole genome sequencing identified mutations in genes related to the electron transport chain (ETC) and respiration (**Extended Data Fig. 4a, b**). However, subsequent testing of strains with deletions of the individual identified genes revealed no significant changes in MIC (**Extended Data Fig. 4c, d**), suggesting that alterations in aerobic respiration/ETC may influence LAR uptake, but the corresponding gene products were unlikely its direct targets. In support of this conclusion, LAR exhibited a 4-to-8-fold increase in MIC against *E. coli* under anaerobic conditions (**Extended Data Fig. 5a**).

We reasoned that our inability to isolate LAR-resistant mutants with alterations in the antibiotic target site could be explained if LAR were acting upon the ribosome because the redundancy of rRNA genes in bacterial genomes (e.g., 10 *rrn* alleles in B. *subtilis* or 7 rrn alleles in *E. coli*) prevents direct isolation of target site mutations. Therefore, we turned to *E. coli* SQ110DTC, which harbors a single rRNA operon and has been successfully used for isolating mutants resistant to ribosome-targeting antibiotics^21,22^. Resistant clones appeared with the frequency of ~ 2×10^−8^ when *E. coli* SQ110DTC cells were plated on LAR-supplemented agar. Sequencing the rRNA operon revealed six types of mutants, all with mutations in the 16S rRNA: three mutants had single nucleotide substitutions (U1052A, G1207A, and C1209G), two represented single nucleotide deletions (ΔG1048 and ΔG1050), and one clone harbored simultaneously the U961C substitution and the deletion of C1200 (**Extended Data Fig. 6a**). All the affected nucleotides are con ned to helices h31 and h34 of the 16S rRNA (**Extended Data Fig. 6b**) and are spatially close to each other in the 3D structure of the small ribosomal subunit (**Extended Data Fig. 6d**). These results reveal the ribosome as the most likely intracellular target of LAR in bacteria. Supporting this conclusion, LAR readily inhibits the expression of reporter proteins in an *E. coli in vitro* transcription-translation-coupled system programmed with DNA or RNA (Fig. 2e, f). The submicromolar IC_50_ of LAR argued that it binds with high affinity to the bacterial ribosome. In contrast, the IC_50_ of LAR in a mammalian cell-free translation system is ~58-fold higher (Fig. 2g), revealing LAR as a selective inhibitor of bacterial protein synthesis. Altogether, our genetic and biochemical experiments showed that, unlike other known LPs, LAR inhibits bacterial growth by targeting the ribosome.

### Lariocidins bind to a distinct site in the small ribosomal subunit.

**Figure 3:**
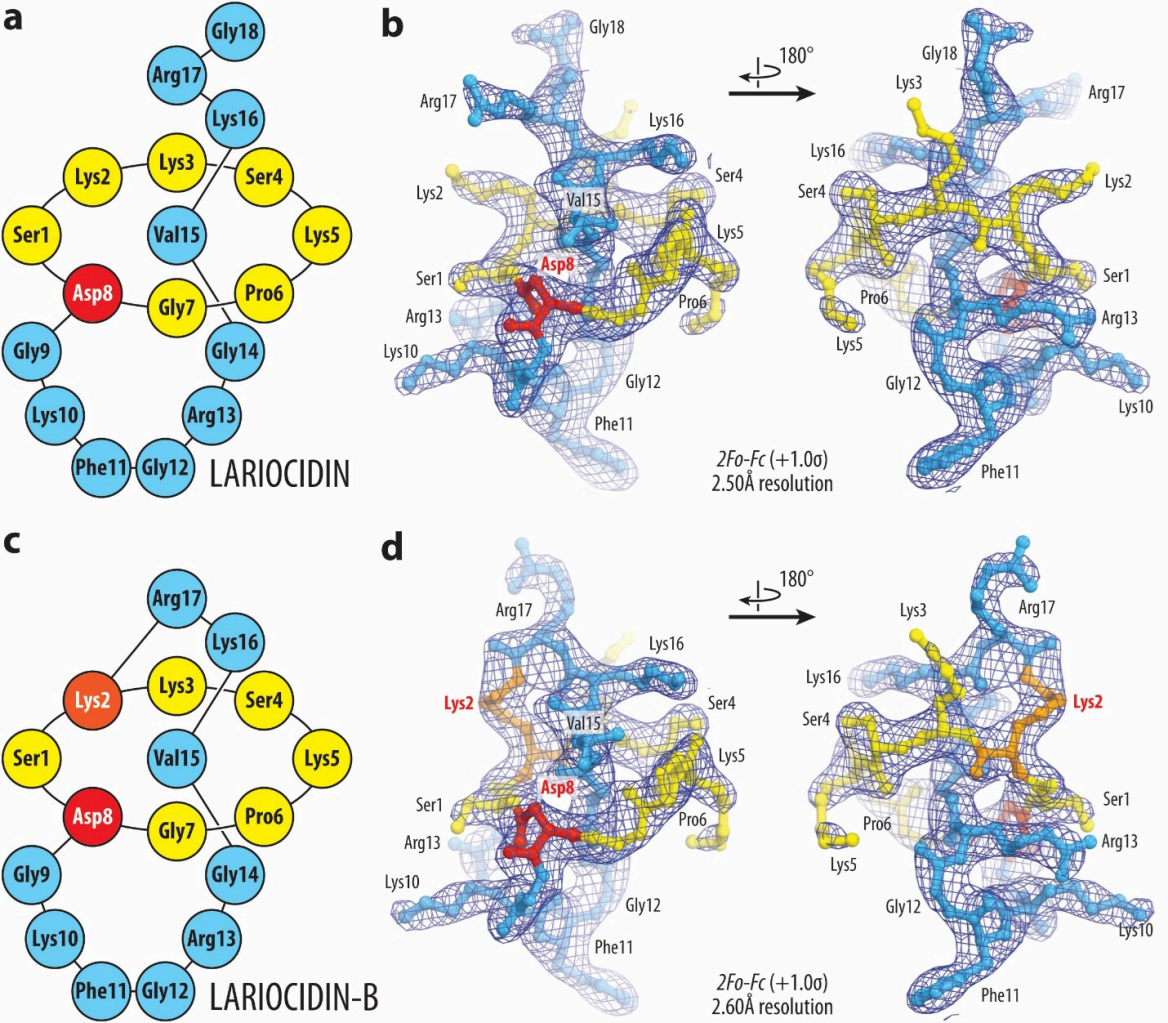
Structures and electron density maps of ribosome-boundLAR-A and LAR-B. **(a, c)** Schematic diagrams of the lasso peptides LAR-A (**a**) and LAR-B (**c**) highlighting their N-terminal residues 1–7 (yellow), branching point at residue 8 (red), and C-terminal residues 9–18 (blue). Lys2 residues of LAR-B forming the second isopeptide bond is colored orange. (**b, d**) 2Fo-FcFourier electron density maps of LAR (**b**) and LAR-B (**d**) in complex with the *T. thermophilus* 70S ribosome (blue mesh). The refined models of lariocidins are displayed in their respective electron density maps after the refinements contoured at 1.0σ. Color scheme as in panels (**a**) and (**c**), respectively.

**Figure 4:**
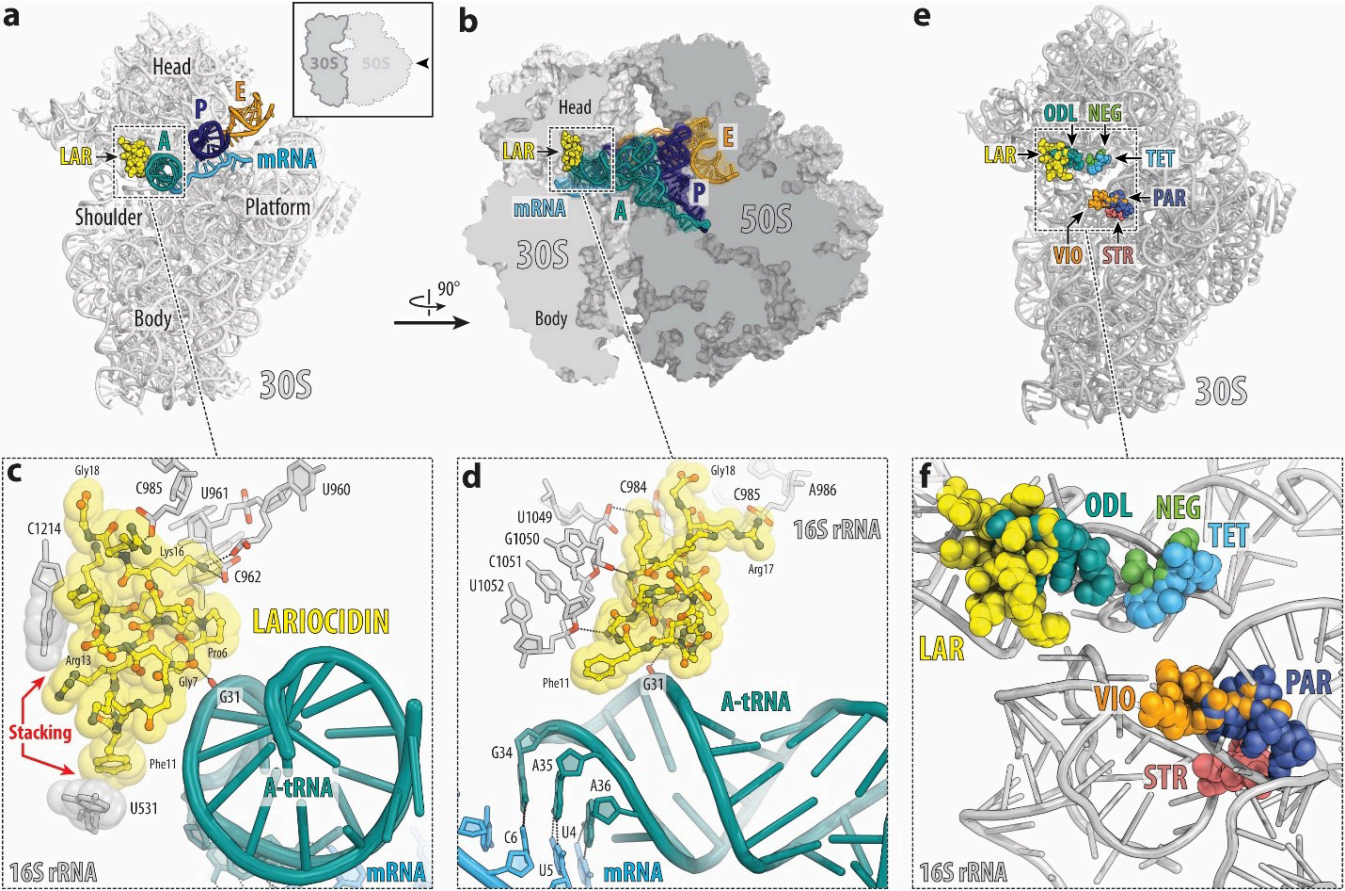
Structure of LAR in complex with the *T. thermophilus* 70S ribosome. **(a, b)** Overview of the LAR binding site (yellow) in the bacterial ribosome, viewed from two different perspectives. The 30S subunit is shown in light grey, the 50S subunit is dark grey, the mRNA is cyan, and the A-, P-, and E-site tRNAs are colored teal, blue, and orange, respectively. In (**a**), the 30S subunit is viewed from the inter-subunit side, as indicated by the inset (the 50S subunit and parts of the tRNAs are removed for clarity). The view in panel **b** is a cross-cut section through the nascent peptide exit tunnel. (**c, d**) Close-up views of the LAR’s interactions with the small ribosomal subunit. The *E. coli*numbering of the 16S rRNA nucleotides is used. H-bonds between LAR, rRNA, and A-site tRNA are indicated with dashed lines. In (**d**), the mRNA nucleotides are numbered relative to the first nucleotide of the P-site codon. (**e, f**) Superposition of structures of antibiotics binding in the vicinity of the decoding center on the small ribosomal subunit. Overview (**e**) and close-up view (**f**) of the ribosome-bound LAR (yellow) relative to the binding sites of other antibiotics targeting the A site of the small ribosomal subunit: odilorhabdin (ODL, teal), tetracycline (TET, blue), negamycin (NEG, green), streptomycin (STR, light red), paromomycin (PAR, dark blue), viomycin (VIO, orange).

To elucidate the interactions of lariocidins with the target, we obtained complexes of LAR or LAR-B with the ribosome from the thermophilic Gram-negative bacterium *Thermus thermophilus (Tth)* bound to mRNA and carrying A-, P-, and E-site tRNAs, crystallized them and determined X-ray crystal structures. At the overall resolution of 2.5 Å and 2.6 Å (for LAR and LAR-B, respectively) (**Supplementary Table 2**), we could unambiguously model all amino acid residues for both LP isoforms (Fig. 3a–d). Both LAR variants form very similar complexes with the ribosome to which they bind in a compact form, with the characteristic knotted lariat fold stabilized by multiple intramolecular hydrogen (H) bonds (**Extended Data Fig. 7a, b**). The unique second isopeptide bond of LAR-B formed between the C-terminal carboxyl of Arg17 and the side chain amine of Lys2 is clearly visible in the electron density map (Fig. 3d). Due to the formation of this bond, the side chain of LAR-B’s Arg17 is reoriented to occupy the space taken by C-terminal Gly18 of LAR-A (**Extended Data Fig. 7c, d**). Consistent with the location of the 16S rRNA resistance mutations (**Extended Data Fig. 6b, d**), LAR binds to the small ribosomal subunit at a distinct site adjacent to the decoding center, establishing simultaneous interactions with the 16S rRNA and the A-site tRNA (Fig. 4a, b). The antibiotic forms multiple contacts with helices h31, h32, and h34 of the 16S rRNA in the 30S subunit head domain. It also extends towards the shoulder domain, where its Phe11 aromatic side chain forms a π-π stacking interaction with the nucleobase of U531. Notably, most contacts of LAR and LAR-B with the ribosome involve the sugar-phosphate backbone of rRNA (Fig. 4c, d) rather than the nucleobases, explaining the modest effects of the selected rRNA nucleotide substitutions on MIC (**Extended Data Fig. 6a**) and suggesting that LAR should be relatively impervious to resistance arising from mutations at the binding site.

In addition to the multiple contacts of LAR with the 16S rRNA, two of its residues (Pro6 and Gly7) closely approach the anticodon loop of the A-site tRNA (Fig. 4c, d). Therefore, we wondered whether the presence of tRNA was essential for antibiotic binding. To address this question, we solved the structure of LAR bound to the *Tth* ribosome lacking mRNA and tRNAs. Despite the absence of tRNAs, we readily observed the electron density attributable to LAR. At 2.6 Å resolution, we could not detect any changes in the overall antibiotic position on the ribosome or its contacts with the 16S rRNA (**Supplementary Fig. 12**) and, therefore, concluded that the presence of A-site tRNA is not essential for LAR binding to the ribosome. However, as discussed below, the LAR-tRNA interactions could be meaningful for certain aspects of the antibiotic action.

The LAR binding site is clearly distinct from the sites of action of other antibiotics targeting the small ribosomal subunit, including odilorhabdins, tetracyclines, aminoglycosides, tuberactinomycins, and negamycin^14,23^ (Fig. 4e, f). Only odilorhabdins^24^ partially encroach upon the LAR binding site (Fig. 4e,f). However, out of 10 known odilorhabdin-resistance mutations^24^, only three, which are identical to the LAR-resistance mutations selected in this study, conferred a 2–4x increase in the LAR MIC (Extended Data Fig. 6c). Collectively, our structural and microbiological data show that LAR displays a unique mode of interaction with the ribosome and shares minimal target-based cross-resistance with clinically relevant antibiotics targeting the small ribosomal subunit.

The location of the LAR binding site and the details of its interaction with the ribosome offer important insights into the mechanism of its action. Because of the contacts with the head and the shoulder domains of the 30S subunit, LAR is expected to restrict the conformational transitions within the subunit required for moving mRNA and tRNAs through the ribosome in the process of translocation^25–27^. To test this prediction experimentally, we assembled a pre-translocation complex (consisting of *E. coli* ribosomes associated with a model mRNA, deacylated tRNA^Met^ in the P site, and *N*-acetyl-Phe-tRNA^Phe^ in the A site) and used primer extension to follow ribosome translocation upon addition of EF-G and GTP in the absence or presence of LAR (Fig. 2h). As expected, in the absence of inhibitors, EF-G/GTP stimulated ribosome translocation by one codon, whereas this reaction was inhibited when LAR was present (Fig. 2h). This result demonstrates that LAR acts as an inhibitor of the translocation step during the elementary protein synthesis cycle. This conclusion was further corroborated by the general toeprinting analysis, which detects the location of antibiotic-stalled ribosomes on mRNA during cell-free translation^28^. We observed that LAR-bound ribosomes were predominantly arrested at the start codon of a model mRNA but also stalled at internal codons of the ORF (Fig. 2i). This pattern is consistent with LAR being an inhibitor of translation elongation. Together, these results reveal LAR as a translocation inhibitor that interferes with the EF-G-catalyzed movement of mRNA and tRNAs through the ribosome.

The direct contact of LAR not only with the ribosome but also with the phosphate of the G31 residue of the A-site tRNA, observed in the X-ray structure (Fig. 3d, e), made us wonder whether this interaction, which is expected to increase the affinity of aminoacyl-tRNA for the ribosome, would render translation error-prone, as observed with other antibiotics that interact simultaneously with the ribosome and tRNA^24,29^. To check if LAR promotes miscoding, we spotted it on a lawn of *E. coli* cells harboring a *lacZ* gene with a nonsense mutation C1456T^30^. The blue halo that appeared on the indicator plate showed that LAR induces premature stop codon readthrough, allowing for the synthesis of the full-length b-galactosidase, thereby substantiating the miscoding activity of LAR (Fig. 2j). Similar to cidal aminoglycoside antibiotics, error-prone translation induced by LAR is a possible cause of its bactericidal activity observed in our early experiments (Fig. 2a).

### LAR exhibits broad-spectrum antibacterial activity and is active in an animal model of infection

Most intracellularly-acting antimicrobial RiPPs, including LPs, are characterized by narrow-spectrum antimicrobial activity due to their reliance on specific peptide importers for entering the cell^31–33^. In contrast, the dependence of LAR uptake on the membrane potential implies a more universal internalization mechanism (**Extended Data Fig. 5**), indicating that LAR may have a broader antimicrobial activity. Indeed, LAR readily inhibits the growth of typical Gram-positive (B. *subtilis*, 1 μg/mL) and Gram-negative (efflux impaired *E. coli*, 2–4 μg/mL) bacteria, as well as mycobacteria (*M. smegmatis*, 1–2 μg/mL) (Table 1). Notably, some clinical isolates of *Acinetobacter baumannii*, a notorious ESKAPE pathogen, including multidrug-resistant and carbapenem-resistant strains, were even more susceptible to LAR, exhibiting MIC in the range of 8–128 μg/mL (Table 1). LAR showed no antifungal action against *Candida albicans* and no activity vs. common anaerobe components of the human microbiome (Table 1). The activity of LAR was significantly enhanced (up to a 128-fold MIC reduction) in nutrient-limited media (Table 1; **Supplementary Table 3**), which better mimic *in vivo* conditions. At the same time, LAR did not display hemolytic activity or cytotoxicity against human cells (Table 1).

**Figure 5:**
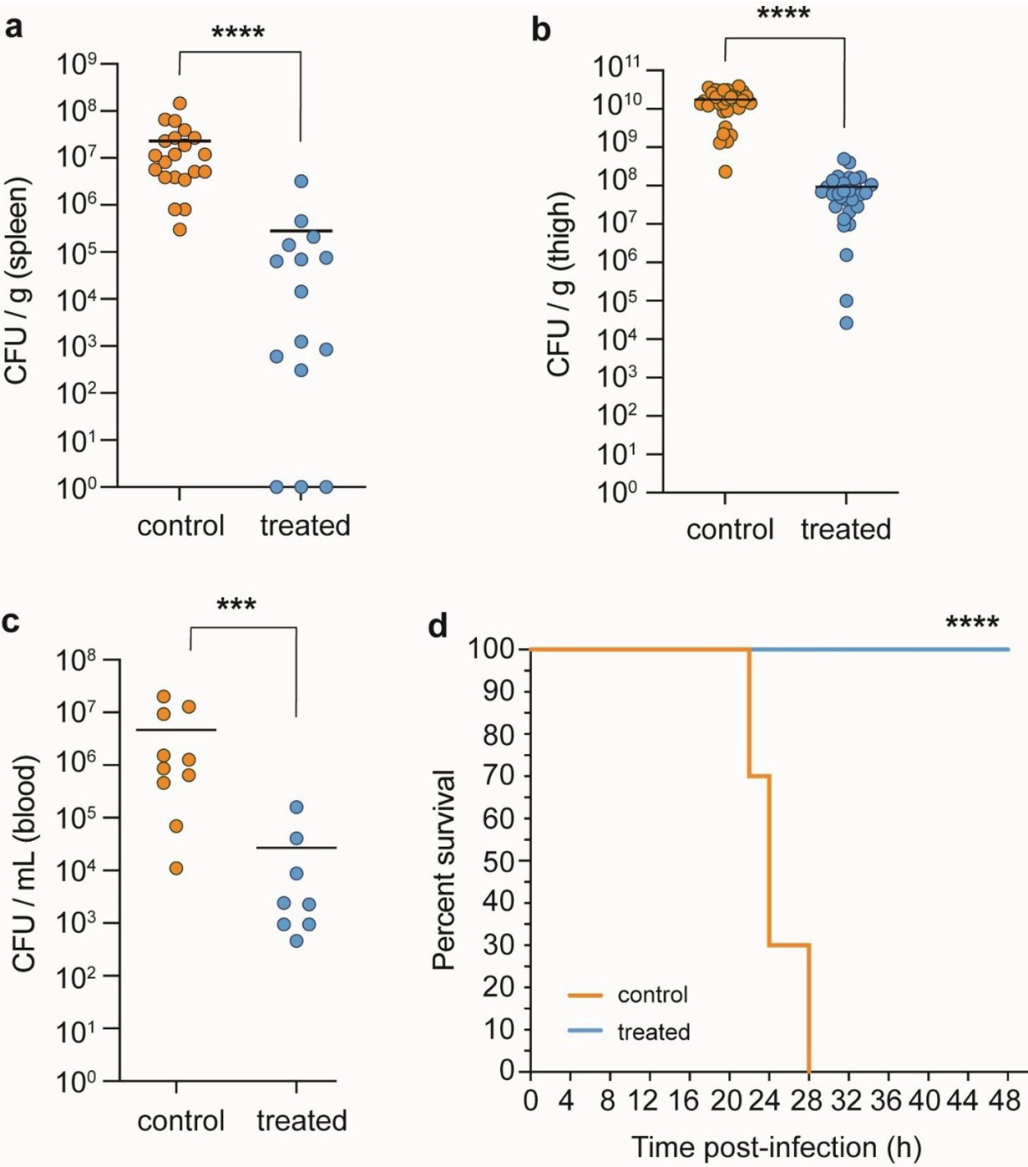
Therapeutic efficacy of Lar in a mouse neutropenic thigh infection model. (**a-c**) Reduction in bacterial burden (*A. baumannii* C0286) after 24 hr post administration of LAR as measured by colony forming units (cfu) per gram of tissue or per mL of blood. (**a**)Bacterial burden in the spleen. control (n = 21) and treated (n = 15) groups. (**b**)Thigh bacterial burden in control (n = 32) and treated (n = 30) groups. (**c**)Blood bacterial burden in control (n = 10) and treated (n = 8) groups. Data points are from individual animals and horizontal lines represent the group means. Significance was determined with a two-tailed Mann-Whitney test (*P≤0.05; **P<0.01; ***P<0.001;****P<0.0001). (**d**) Kaplan-Meier test showing group survival across select time points throughout *A. baumannii* thigh infection in vehicle control and LAR-treated mice. ****P <0.0001, Log-rank (Mantel-Cox) test.

Encouraged by these findings, we tested LAR’s antibiotic activity in a neutropenic mouse thigh-infection model. Treatment of mice infected with a clinical MDR strain of *A. baumannii* resulted in a significant (2 orders of magnitude) reduction in bacterial burden in the spleen, thigh, and blood 24 hrs after administering LAR compared with the vehicle control (Fig. 5a, **Supplementary Table 4, Extended Data Fig. 8**). Moreover, while none of the mice in the control group survived past 28 h, 100% of the LAR-treated mice survived over 48 h post-infection, and appeared much healthier than control mice (Fig. 5b, c). These results highlight the potential of LAR as a promising scaffold for further development into a clinical antibiotic for the treatment of serious bacterial MDR infections.

### LAR-like LPs are encoded in genomes of diverse bacteria

Given the LAR’s potency against clinically relevant pathogens and its unique mechanism of action among LPs, we wanted to explore the naturally occurring diversity of LAR-like compounds produced by bacteria, which could fuel further advancement of LAR as a therapeutic agent. A bioinformatic search for proteins similar to the LrcC lasso cyclase in available bacterial genomes showed that bacteria as diverse as Alpha- and Gamma-proteobacteria, Bacilliota, and Actinomycetota harbor *lrc*-like BGCs in their genomes (**Extended Data Fig. 9a**). The structurally and functionally critical amino acid residues of LAR, including the isopeptide bond-forming Asp, its neighboring Gly residues as well as positively charged amino acids, were highly conserved in precursor peptides encoded in these BGCs (**Extended Data Fig. 9b**). Notably, the clade of *lrc*-like BGCs in Actinomycetota includes the cluster of the previously identified antimycobacterial LP triculamin^34^, whose core peptide differs from that of LAR in only five positions. This similarity suggests that, like LAR, triculamin, whose mode of action remains unknown, likely targets the ribosome. Our limited genome mining analysis shows a wide distribution of *lrc*-like BGCs and provides the basis for further optimization of LAR through mutagenesis and/or chemical modification.

## DISCUSSION

Here, we report the discovery of LAR – a new LP that represents the first in its class antibiotic that inhibits bacterial growth by binding to the ribosome, resulting in mistranslation and interfering with protein biosynthesis by impairing translocation. LAR is the only RiPP known to target the 30S ribosomal subunit, to which it binds in a unique way. The location of the LAR binding site and its specific interactions with the distinct moving domains of the small ribosomal subunit, as well as with the A-site tRNA, account for the dual mechanism of action. Likely by restricting the structural rearrangements of the 30S subunit, LAR inhibits EF-G-catalyzed translocation, whereas its interaction with aminoacyl-tRNA is possibly the primary cause of its miscoding activity. Due to its idiosyncratic structure and binding mode, LAR is mostly insensitive to mutations or enzymatic resistance mechanisms that cause high levels of resistance to the other antibiotics targeting the ribosome (**Extended Data Fig. 10**). While a potent inhibitor of bacterial translation, LAR has only a marginal effect on eukaryotic translation, which is consistent with a lack of cytotoxicity.

With its second isopeptide bond that forms a double lariat, one of the lariocidin isoforms, LAR-B, is unique among all known LPs and should be considered the founder of a new class (Class V) of LPs. While the activity of LAR-B closely matched that of LAR, we expect that the second amide linkage may even further increase thermodynamic and metabolic stability, the known general features of the LPs, which may be a valuable asset for future drug development. The high positive charge of lariocidins distinguishes them from most other intracellularly acting RiPPs. Similar to the internalization of the positively charged aminoglycosides, LAR’s uptake is likely transporter-independent and, instead, mediated by the membrane potential, explaining its broad antimicrobial spectrum. Similarity with the clinically successful aminoglycosides extends even further - LAR is bactericidal, likely due to the miscoding activity.

The beneficial physico-chemical and microbiological features of LAR and its demonstrated efficacy in an A. *baumannii* infection model make it a promising candidate for advancement toward a clinically useful drug. The ability to express the *lrc* BGC in a heterologous host, the clear understanding of interactions of LP with the ribosome, and the knowledge of the variety of LAR-like LPs encoded in the bacterial genomes open obvious chemical and biological routes for further optimization of LAR and other ribosome-targeting LPs. Developing these new antibiotics with their low propensity for resistance offers hope for a new class of drugs, much needed to address the antibiotic resistance crisis.

## ONLINE METHODS

### Isolation of strains and screening for antimicrobial activity

One gram of soil (from Hamilton, Canada) was mixed with 10 ml of PBS and serially diluted before being plated on a soil agar medium. To prepare soil agar, 100 g of soil was mixed with 700 mL of MilliQ water, shaken for several hours, and centrifuged to remove insoluble particles. The supernatant was mixed with 1.5% agar and autoclaved. Soil/agar plates were incubated at 30°C. Fast-growing colonies were picked over the first 3–4 weeks. To isolate slow-growing colonies, plates were wrapped in plastic (tube bag used for petri dish packaging, SARSTEDT) to prevent dehydration and stored at room temperature (RT) for one year. Colonies that appeared were streaked on fresh Tryptone Soy Agar (TSA; Fischer Scientific) plates. From these plates, 80 colonies were isolated and tested for antibacterial activity by the following procedure. Strains were grown in 10 mL of various nutrient media (Tryptone Soy Broth (Fischer Scientific), half-strength Brain Heart Infusion broth (Fischer Scientific), Davis Minimal Broth (Sigma-Aldrich) with 0.5% peptone (Gibco^™^ Bacto^™^)), and crude methanolic extracts were prepared by lyophilizing 1 mL of cell-free supernatant at different intervals over the 10 days. Extracts (10 μL) were spotted on cation-adjusted Mueller-Hinton Agar (MHA; BD Difco^™^) plates containing lawns of the tester strains A. baumannii C0286 and *E. coli* BW25113Δ*tolC*Δ*bamB*.

### Fermentation and purification of LAR

The *Paenibacillus* M2 isolate consistently showed bioactivity in tryptic soy broth (TSB) medium against both tester strains. Early attempts to purify LAR from cells grown in TSB were unsuccessful, partly owing to low yield, prompting optimization of fermentation conditions. Replacing glucose with 10 g/L starch and casein enzyme hydrolysate with casamino acid (17 g/L) in TSB generated CSB (Casein Starch Broth) medium. In CSB, LAR production reached 10–15 mg/L. For LAR purification, the *Paenibacillus* M2 inoculum, prepared overnight in 100 mL CSB, was diluted 100-fold in 1 L of sterile CSB in a 2.8 L Fernbach Flask and incubated with shaking (200 rpm) for 21–23 h at 30°C. Cells were removed by centrifugation and the cell-free supernatant was treated with pre-activated Diaion HP20 resin (5% v/v) for 2–2.5 hours. Resin was washed with 20% MeOH and adsorbed metabolites were eluted with 100% MeOH. The MeOH extract was dried under vacuum, dissolved in water, and fractionated using an SP-sepharose cation-exchange chromatography. The column was pre-equilibrated with 10 mM ammonium acetate buffer (Buffer A; pH 5.0–5.2). Sample pH was also adjusted in a similar range and loaded through a peristaltic pump. The unbound sample was washed with Buffer A, and elution was performed with 0.5 M, 0.75 M, and 1.0 M NaCl in Buffer A, with the 0.75 M fraction containing LAR. This fraction was further purified using a C18 column on a CombiFlash system (Teledyne Inc.), followed by preparative reversed-phase high-pressure liquid chromatography (RP-HPLC) using a C8 column (Eclipse XDB-C8 SemiPrep 9.4×250, 5μM, Agilent Technologies), yielding LAR as a single peak with >95% purity, confirmed by analytical HPLC. Acetonitrile/water containing 0.07% trifluoroacetic acid (TFA) was employed as the mobile phase for both CombiFlash and HPLC systems. The compound was lyophilized to a white fluffy TFA salt and re-lyophilized with diluted HCl to remove TFA.

### Structural characterization of LAR

The chemical characterization of LAR was performed using a combination of mass spectrometry, Marfey’s amino acid analysis, and NMR spectroscopy. Mass spectrometry included Liquid Chromatography-Electrospray Ionization-High Resolution Mass Spectrometry (LC-ESI-HRMS or simply LC-MS), Matrix-assisted Laser Desorption/Ionization mass spectrometry (MALDI-MS,) and MALDI-MSMS. LC-ESI-HRMS data were acquired using an Agilent 1290 UPLC separation module and qTOF G6550A mass detector in positive-ion mode. MALDI-MS and MSMS data were obtained on Bruker Ultra eXtreme MALDI TOF/TOF and re ector detector in positive-ion mode. For amino acid analysis, 0.5–1 mg of the compound was hydrolyzed with 6 N HCl at 110°C overnight, derivatized with Marfey’s reagent (Thermo Scientific), and analyzed by LC-ESI-HRMS. NMR analysis was performed with 15 mg of compound dissolved in deuterated D_2_O, and 1D and 2D spectra were recorded on a Bruker AVIII 700 MHz instrument equipped with a cryoprobe.

### Whole genome sequencing and BGC analysis

Genomic DNA from *Paenibacillus* sp. M2 was isolated using a DNA isolation kit (Promega) and sequenced using Nanopore and Illumina MiSeq. Unicycler was used to perform a hybrid assembly of Illumina and Oxford Nanopore reads^35^. SPAdes was first used to generate a short-read assembly graph, which was scaffolded with long reads. Multiple rounds of polishing were conducted using Pilon. A single contig was obtained and analyzed using antiSMASH 6.0^36^ to identify the BGC responsible for LAR synthesis.

### Identication of lrc-like BGCs and Phylogenetic tree construction

To explore the diversity of *lrc*-like BGCs in other bacteria, the LrcC amino acid sequence was used as a query in NCBI BLAST, and the hits were manually checked for redundancy. The genomes of potential hits were analyzed manually and were searched for *lrc*-like BGCs using antiSMASH 7.0^37^. The BGCs were manually curated and analyzed for Lar-like core-peptide sequences. A total of 29 complete BGCs were identified. The LrcC-like cyclases from these BGCs were aligned using the MUSCLE algorithm (default parameters). The aligned sequences were used to construct a maximum-likelihood (ML) phylogenetic tree using MEGA11^38^, using the WAG substitution model, with a bootstrap value of 100, and keeping other parameters as default. The tree was rooted to an unrelated lasso-cyclase from paeninodin BGC as an outgroup. The BGCs from different bacterial species were aligned using clinker^39^ tool and inspected manually for the presence of various genes. The amino acid sequences of core peptides from all BGCs were aligned, and the consensus sequence was derived using JalView v. 2.0^40^ with default parameters.

### Heterologous expression of LAR

To express LAR in a heterologous host, all the genes in LAR BGC (*lrcA to lrcF*) were codon optimized for *Streptomyces lividans* using GenScript’s optimization tool, and the BGC was synthesized as three GBlocks with sequence homology to each other and to the vector, incorporating synthetic promoter and ribosome-binding sequences (RBSs). The fragments were combined and integrated into the plasmid pIJ10257^41^, digested with NdeI and KpnI, via Gibson assembly^42^. The assembly mixture was transformed into *E. coli* TOP10 electrocompetent cells. Plasmids from selected colonies were analyzed via restriction mapping and further validated by sequencing through Oxford Nanopore technology (Plasmidsaurus). A correct plasmid, designated pIJ10257-*lrc* (**Supplementary Data Fig. 10**), was transformed into *E. coli* ET12567 electrocompetent cells, creating the strain ET12567 pIJ10257-*lrc*.

To move the plasmid into the heterologous Streptomyces lividans host, we performed triparental conjugation using ET12567 pIJ10257-lrc as the donor strain, ET12567 pR9406 as the helper strain, and spores of *Streptomyces lividans* XF10 (a derivative of *S. lividans* TK24 strain) as recipients. The *E. coli* strains were cultured overnight in LB medium with appropriate antibiotics, then subcultured to mid-exponential phase (OD_600_ 1.0–1.2). Cells were harvested, washed, and mixed with activated *Streptomyces* spores. This mixture was plated on mannitol-soyflour agar (MSA; g/L: mannitol (20), soyflour (20), and agar (20)) plates and incubated at 30°C overnight. The next day, the plates were overlaid with 1 mL of water with nalidixic acid and hygromycin (final concentration in agar plate as 25 μg/ml and 50 μg/ml, respectively) to eliminate *E. coli* and to select for exconjugants containing the *pIJ10257-lrc* plasmid. The resulting *Streptomyces* strain was termed SL-Lar. To delete the lrcF gene, the pIJ10257-lrc was used as a template for PCR amplification using the primers LrcΔF-F1, -F2, -R1, and -R2 (**Supplementary Table 1**) to yield two fragments. The resulting fragments were assembled and successfully integrated into S. lividans, as described above, to yield *S. lividans* pIJ10257-*lrcΔF* (SL-LarΔF) strain.

Three exconjugants for each strain were chosen for fermentation and LAR analysis. These exconjugants were grown in manually designed antibiotic-screening medium (ASM; composition (g/L); starch (10), casein enzyme hydrolysate (10), ammonium sulfate (10), MgSO_4_.7H_2_O (1), NaCl (5), CaCO_3_ (0.5), KH_2_PO_4_ (0.7), K_2_HPO_4_ (0.9)) for four days. Crude extracts were prepared using Diaion HP20 resin and subjected to cation-exchange chromatography as described above. The fractions were analyzed by RP-HPLC, and the chromatograms were compared to LAR purified from *Paenibacillus* sp. M2, with peak identity confirmed by mass spectrometry.

### MIC determination

MICs were determined using broth microdilution method in cation-adjusted Mueller-Hinton Broth (MHBII, BD Difco) following standard procedures unless stated otherwise. *Mycobacteria* susceptibility was tested in Middlebrook 7H9 medium supplemented with 10% OADC Enrichment (oleic acid, bovine albumin, dextrose, and catalase) (BD Difco). The compounds (2 to 4 μl) were mixed with diluted cultures (5–7 × 10^5^ cfu/mL) in MHB (196 to 198 μL) in 96-well round-bottom plates, and 2-fold serial dilutions were made. Plates were incubated at 37°C for 18–22 h, and OD_600_ readings were taken on Synergy Microplate Reader (Biotek). MIC was determined to be the lowest concentration of antibiotics with no visible growth. For Mycobacterial cultures, the plates were read after two to three days. Susceptibility was also assessed in MOPS minimal medium with 0.4% glucose (M2106 Teknova) and RPMI-1640 medium. Serum effects were tested in MHB with 10% or 50% fetal bovine serum (Invitrogen) or human serum (Heat Inactivated, from human male AB plasma; Sigma-Aldrich). For anaerobic conditions, *E. coli* was incubated in BD GasPak^™^ EZ pouch systems. To adjust the pH of the media, 1M NaOH or 1N HCl was used.

For MIC testing using human microbiota strains, the cultures were grown in BHI supplemented with L-cysteine (0.5g/L), Hemin (10mg/L), and Vitamin K (1mg/L) in anaerobic chambers (37 °C, 5% H_2_, 10% CO_2_, 85% N_2_). *Candida albicans* was cultivated in yeast nitrogen base (YNB) medium without amino acids (BD Difco).

To determine the cross-resistance of LAR with other translation inhibitors, MIC was measured in *E. coli* BW25113 Δ*tolC*Δ*bamB* strain expressing various resistance genes as described elsewhere^43^.

### Cytotoxicity of LAR against human cells

HEK293 cells were seeded at 7500 cells/well in 384-well plates with DMEM supplemented with 10% FBS, 2 mM L-glutamine, 100 U/mL penicillin, and 100 μg/mL streptomycin, incubated for 18 h at 37°C in the atmosphere of 5% CO_2_. DMSO-dissolved compounds or DMSO were added using a Labcyte Echo acoustic dispenser (Beckman Coulter) and a combi nL (ThermoFisher) dispenser, and cells were incubated for 48 h. Cell viability was assessed using Promega Cell Titer Glo 2.0 reagent, with luminescence read on a Neo2 plate reader (Biotek). Wells with no solvent added, or those supplemented only with DMSO were used as controls. A prestoBlue cell viability assay was also used to evaluate cell survival and assess cytotoxicity.

### Hemolysis

Human blood (BioIVT, USA) was centrifuged to remove plasma, and erythrocytes were washed with 0.85% NaCl and resuspended in PBS (pH 7.4) to maintain hematocrit. Compounds (1 μl) were added to a 96-well V-bottom plate using Labcyte Echo, with 1% DMSO (final concentration) as a control. Triton X-100 served as a positive control. Erythrocytes were diluted 1:50 in PBS, 99 μl added to wells, incubated at 37°C for one hour, centrifuged at 1000 xg for 5 min, and the optical absorbance of the supernatant was measured at 540 nm on a Neo2 plate reader (Biotek). The release of hemoglobin with Triton-X was considered 100% hemolysis.

### Time-dependent killing and cell lysis assay

*E. coli* BW25113 was grown overnight in 5 ml MOPS minimal medium and diluted 1:100 in 3 ml of fresh medium in 14 ml culture tubes. Cells were grown for three to four hours to reach mid-exponential phase (OD_600_ 0.4–0.6) and treated with 10xMIC of LAR (40 μg/ml) or colistin (2.5 to 5 μg/ml) in 1 ml culture at 37°C with agitation at 250 rpm. Cultures were sampled at various times, centrifuged at 10,000 xg for 3 min, and plated on MHA plates. The number of colony forming units (cfu) was determined after incubating plates for 20–24 hr at 37°C. A time-kill assay against A. *baumannii* C0286 was performed in MHB. For cell lysis assay, cultures were prepared as described above. Initial OD_600_ was adjusted to 0.15–0.2, and 98 μl of culture was added to a 96-well plate containing 2 μl of compound. The plates were incubated with orbital shaking at maximum speed with a Synergy Microplate Reader (Biotek), and OD_600_ was measured over 6 h. Experiments were performed in two to three biological replicates. For colistin in cell lysis assay, clumping of cells was observed after ≈2h, leading to a false increase in OD; therefore, data for colistin was not plotted after this period. The plate was manually mixed on a vortex, and the turbidity of colistin-treated wells was confirmed.

### Propidium iodide uptake assay

*E. coli* BW25113 cells were prepared as described above and mixed with propidium iodide (final concentration 4 μM). Cell suspension (190 μL) was then placed into wells of a 96-well black-wall plate and supplemented with 10 μL of compounds at various concentrations. Fluorescence (lex/lem: 535/617 nm) was monitored for 30 min at RT in a Synergy Microplate Reader (Biotek). Colistin was used as a positive control.

### b-galactosidase activity assay

*E. coli* TOP10 cells with pUC19 plasmid (containing the *lacZ* gene) were grown to mid-log phase, OD600 adjusted to 0.1, and treated with Ortho-nitrophenyl-β-D-galactopyranoside (ONPG) (1.5 mM). Cells were mixed with compounds in a 96-well plate (final volume 200 μl), incubated at 37°C, and absorbance at 420 nm measured for 30 minutes. Colistin was used as a control.

### Membrane depolarization assay

Mid-exponential phase *E. coli* BW25113 culture (OD_600_ 0.3–0.5) was mixed with 3,3′-Dihexyloxacarbocyanine iodide (DiOC_2_(3), ThermoFisher Scienti c) dye at a nal concentration of 30 μM and 190 μL of cells were placed into wells of a 96-well black plate. A volume of 10 μl of test compounds or DMSO was added, mixed by pipetting, and fluorescence was measured for 60 min at room temperature using a Synergy Microplate Reader (Biotek) (lex/lem: 450/670 nm). Carbonyl cyanide m-chlorophenyl hydrazone (CCCP) served as a positive control.

### Scanning electron microscopy

Approximately 10^8^ cells of *E. coli* BW25113 were treated with LAR (40 μg/ml, 10xMIC) in MOPS minimal medium for 1 h at 37°C, centrifuged at 5,000 xg for 5 min and resuspended in 0.1x volume of fixative solution (4% glutaraldehyde in PBS, pH 7.4). Cells were fixed at room temperature for 1 h and stored at 4°C overnight. The next day, 50 μL of the fixed cells were placed on poly-L-lysine coated coverslips, dehydrated through graded ethanol steps, and dried using a critical point dryer. Samples were analyzed using a scanning electron microscope (TESCAN VEGA-II LSU) equipped with an X-MAX 80mm2 EDS detector, and images were acquired using INCA software.

### Selection of spontaneous resistant mutants

Approximately 10^9^ cfu of *E. coli* BW25113 or *B. subtilis* 168 were plated on MOPS minimal agar and Mueller-Hinton agar, respectively, containing varying concentrations of LAR. Plates were incubated at 37°C for 24–48 h. Colonies that appeared were tested for LAR susceptibility using MIC assays. Genomic DNA from representative resistant mutants (n=3 for *E. coli* and n=6 for *B. subtilis*) was isolated and sequenced using Illumina technology. The resulting sequences were compared to the reference genome of the parental strains to identify mutations using breseq^44^.

To identify LAR-resistance mutations in rRNA genes, *E. coli* SQ110 *ΔtolC* (SQ110DTC)^45^ harboring a single *rrn* operon was grown overnight in MHB supplemented with 50 mg/mL of kanamycin. Cells were diluted 100-fold into fresh MHB grown until cell density reached OD_600_ of 0.6. Then, 1 OD_600_ unit of cell culture (~0.85 × 10^9^ cells) was plated on an MHB/agar plate containing 50 μg/mL kanamycin and 64 μg/mL LAR (~4xMIC). Several dozen colonies appeared after 48 h incubation at 37°C. For 20 randomly selected colonies, rDNA was PCR-ampli ed using the primers rrnE_F and rrnE_R (**Supplementary Table 1**) and sequenced. The LAR MIC in liquid MHB medium was then determined for the isolates harbouring mutations in the rDNA (n=8).

### Synthesis of LAR-fluorophore conjugates and purification of LAR-B

To protect the free amines in LAR, di-*tert*-butyl decarbonate (Boc anhydride) was selected as the protecting group. LAR (30 mg, 0.016 mmol, 1 eq) was dissolved in 16 ml of 50% acetonitrile/water. After addition of Boc-anhydride (35 mg, 0.16 mmol, 10 eq) and triethyl amine (TEA, 32.3 mg, 45 μl, 0.32 mmol, 20 eq), the reaction mixture was stirred at room temperature (RT) for 1 h. The reaction was confirmed by LC-ESI-HRMS for the presence of Boc groups on LAR. Two major products were identified as 1186.29 [M+2H]2+ and 1098.74 [M+2H]2+, corresponding to penta-Boc-protected LAR (Boc(5)-LAR) and tetra-Boc-protected LAR-B (Boc(4)-LAR-B) respectively. A small peak corresponding to Boc(5)-LAR-C was also detected. The crude mixture was lyophilized to yield compound **2** (Boc-LAR mix).

To attach a click-chemistry handle on the free carboxyl group of the C-terminus, propargyl amine was selected and amidated as follows: Compound **2** (30 mg, 0.013mmol) was dissolved in 1 mL 50% DMSO/DMF, and to this solution were added propargyl amine (3.6 mg, 0.065mmol, 5eq), TEA (13.1 mg, 0.13 mmol, 10 eq) and PyBop (33.8 mg, 0.065 mmol, 5 eq) as 1 M solutions in 50% DMSO/DMF. The reaction was stirred at RT for 30 min, analyzed by mass spectrometry, and then purified using reversed-phase chromatography (C18, 50 g) on a CombiFlash system (Teledyne Inc.). Water/acetonitrile containing 0.07% TFA was used as the mobile phase. Boc(4)-LAR-B did not react with propargyl amine in the above reaction, indicating that C-terminal carboxyl is not free. The peak corresponding to this compound was resolved from Boc(5)-LAR and Boc(5)-LAR-C on a C18 column (owing to the differences in their hydrophobicity), collected separately and processed as described below to yield pure LAR-B. The other two compounds reacted with propargyl amine successfully and eluted as a single peak that was collected and lyophilized to yield compound **3** (Boc-LAR-alkyne). Boc-deprotection of compound **3** was performed in 5 ml of 20% TFA in DCM at RT for 20 min. The solvent was evaporated under nitrogen gas, resuspended in 50% acetonitrile/water, and lyophilized to obtain compound **4** (LAR-alkyne). Boc(4)-LAR-B was deprotected similarly to yield pure Lar-B. The homogeneity of LAR-B was confirmed by LC-MS (**Supplementary Fig. 11a**).

For conjugation of the BODIPY or rhodamine dyes, click-chemistry^46^ was employed as follows: compound 4 (2 mg, 0.001mmol) was dissolved in 0.45 mL of H_2_O, after which copper(II) sulfate and sodium L-ascorbate were added to the final concentrations of 250 μM and 5 mM, respectively. BODIPY FL azide (Lumiprobe Corporation) or rhodamine azide^46^ (65 μl from 10 mM stock solution in DMSO) were then introduced into the reaction and allowed to proceed for 30 min at RT with stirring. The reaction was monitored by mass spectrometry, and the final product was purified using RP-HPLC to obtain LAR-BODIPY or LAR-rhodamine. The fluorophore-conjugates were lyophilized and dissolved in DMSO for further experiments.

### Fluorescence microscopy

*E. coli* BW25113 was grown overnight in MOPS minimal medium, then subcultured in fresh medium and grown to mid-exponential phase (OD_600_ 0.3–0.4)). Two μL of LAR-BODIPY or LAR-rhodamine (final concentration 20 μg/mL) from a stock solution in DMSO were added to 0.5 mL of cell cultures and incubated for 1–2 h at 37°C. Twenty min before harvesting the samples, Hoechst 33342 dye was added to the final concentration of 20 μg/mL, and 2 min before harvest, FM-4–64 (final concentration 20 μM) was added. Cells were centrifuged to remove excess dye and the compound and resuspended in 25–50 μL of PBS. Five μL of cell suspension were spotted on PBS agarose pads (1% agarose) and covered with high-precision cover glass (1.5H Thickness, cat. no. NC1415511, Fischer Scientific). The slides were visualized using a ZEISS LSM980 confocal microscope (Zeiss), and the images were acquired and processed using ZEN Blue software.

To test the effect of pH on localization, the pH of the MOPS minimal medium was adjusted using 1M NaOH or 1N HCl, and the cells were subcultured in a modified medium until mid-exponential phase before treatment as described above. For the CCCP assay, LAR-BODIPY and CCCP were added simultaneously. All confocal microscopy experiments were conducted minimally in duplicate.

### In vitro transcription/translation assay

The effect of LAR on *in vitro* protein synthesis was assessed using an *E. coli* S30 extract transcription-translation system for circular DNA (Promega), with pBESTluc plasmid DNA serving as the template for the firefly luciferase production. Following the manufacturer’s protocol, the reactions were carried out in 25 μl in a 96-well plate. LAR was tested at concentrations ranging from 0 to 50 μM. Reactions were incubated for one hour at 37°C. Luminescence was measured in a 96-well opaque plate using Synergy Microplate Reader (Biotek). IC_50_ values were calculated using GraphPad Prism 10 software.

To test the effect of LAR specifically on translation, sfGFP mRNA was generated by *in vitro* transcription using MEGAscript^™^ T7 Transcription Kit (Thermo Scientific) and purified by lithium chloride (LiCl) precipitation method as described in the manufacturer’s manual. *In vitro* translation was carried out in the system as mentioned above with real-time monitoring of sfGFP fluorescence (lex/lem: 483nm/513nm) for one hour. 1μl of mRNA (from 1μg/μl stock) was added in a 25 μl reaction.

### In vitro translation in the rabbit reticulocyte lysate

The effect of LAR on eukaryotic translation was assessed using Rabbit Reticulocyte Lysate System (L4960, Promega) programmed with firefly luciferase mRNA. *In vitro* reactions were assembled according to the manufacturer’s protocol in a nal volume of 10 μL supplemented with 0.02–50 μM of LAR or no antibiotic and incubated for 90 min at 30°C. After that, 2.5 μL from each reaction was mixed with 50 μl of prewarmed luciferase assay reagent, and the luminescence was measured in Infinite M200 PRO plate reader (TECAN) for 10 min at 25°C. The assay was performed in triplicate.

### Toeprinting assay

Toe-printing analysis was carried out in the *E. coli in vitro* transcription-translation system assembled from the purified components (PURExpress, NEB) as described previously^47^. Reactions either contained no antibiotic or were supplemented with 50 μM retapamulin or varying concentrations of LAR (1, 5, or 10 μM). The model RST1 template encoding a 21-aa long peptide containing all 20 proteinogenic amino acids^22^ was generated by PCR using the primers RST1_F and FIRST1_R (**Supplementary Table 1**).

### Translocation inhibition assay

*In vitro* translocation assay was carried out with a model mRNA with the sequence 5’- AUUAAUACGACUCACUAUAGGGCAACCUAAAACUUACACACGCCCCGGUAAGGAAAUAAAA-AUG-UUC-AAA-GCA-UUC-AAA-AAC-AUC-AUA-CGU-ACU-CGU-ACU-CUU-UAAGCGCAGGCAAGGUUAAUAAGCAAAAUUCAUUAUAACC - 3’ encoding the MFKAFKNIIRTRTL peptide (underlined part). The mRNA was prepared by *in vitro* transcription of a PCR product amplified using the primers MF_F1, MF_F2, and MF_R (Supplementary Table 1). *In vitro* transcription was performed using HiScribe^®^ T7 High Yield RNA Synthesis Kit (New England Biolabs) as recommended by the manufacturer. A 4.5 μL reaction containing 1 μM *E. coli* ribosomes, 0.5 μM mRNA, 1 μM tRNA_i_^Met^, 0.5 μM radiolabelled NV1 primer (**Supplementary Table 1**), 2 U/μL RiboLock RNase Inhibitor (Thermo), and antibiotic tested (62.5 μM LAR or 250 μM negamycin) in Pure System Buffer (PSB; 9 mM Mg(CH_3_COO)_2_, 5 mM K_3_PO_4_, 95 mM potassium glutamate, 5 mM NH_4_Cl, 0.5 mM CaCl_2_, 1 mM spermidine, 8 mM putrescine, 1 mM dithiothreitol, pH 7.3)^48^ was incubated for 20 min at 37°C. Then N-acetyl-Phe-tRNA^Phe^ was added to the nal concentration of 2 μM followed by 10 min incubation at 37°C. After that, *E. coli* EF-G and GTP were added to the final concentrations of 0.2 μM and 533 μM, respectively. After 5 min incubation at 30°C, 1 μL of the mixture of AMV reverse transcriptase (Roche) and dNTPs (2.1 U/μL AMV RT and 2 mM dNTPs in PSB) was added, and the reactions were incubated for another 5 min at 30°C. To stop the reaction, 200 μL of the resuspension buffer (300 mM NaCH_3_COO, 5 mM EDTA, 0.5% SDS) were added. DNA was then extracted following the phenol-chloroform extraction procedure and precipitated by adding 3 volumes of ice-cold ethanol, incubating at −70°C for 15 min, and centrifugation for 30 min (4°C, 20000 g). The reaction products were resolved in 6% sequencing polyacrylamide gel and imaged on the Typhoon phosphorimager.

### Miscoding assay

*E. coli* strain CA244^30^ harboring a premature stop codon in the *lacZ* gene (C1456T), was used in the *in vivo* miscoding assay. The production of full-length functional β-galactosidase was monitored by the appearance of the blue halo on the indicator agar plates containing X-gal (5-bromo-4-chloro-3-indolyl-β-D-galacto-pyranoside). For the X-gal assay, cells were plated on MOPS agar (Teknova) supplemented with 80 μg/ml X-gal and 250 μM IPTG. Drops of LAR, streptomycin, gentamicin, and chloramphenicol solutions (containing 60.7, 4, 3.1, and 0.9 μg of the antibiotic, respectively) were spotted on the plates. Plates were incubated for ~18 h at 37°C and photographed.

### X-ray crystallographic structure determination

Wild-type 70S ribosomes from *T. thermophilus* (strain HB8) were prepared as described previously^49–52^. Synthetic mRNA with the sequence 5’-GGC-AAG-GAG-GUA-AAA-AUG-UUC-UAA-3’ containing Shine-Dalgarno sequence (underlined) followed by methionine (AUG) and phenylalanine (UUC) codons were obtained from Integrated DNA Technologies (USA). Non-hydrolyzable aminoacylated Phe-tRNA^Phe^ and fMet-tRNA_i_^Met^ were prepared as described previously^51,53–55^.

LAR and LAR-B compounds were soaked into the pre-formed crystals of 70S ribosome in a functional state corresponding to the PTC pre-peptide bond formation with unreacted aminoacylated Phe-tRNA^Phe^ and fMet-tRNA_i_^Met^ in the A and P sites, respectively. Complexes of the wild-type *T. thermophilus* tightlycoupled 70S ribosomes with mRNA, aminoacylated A-site Phe-tRNA^Phe^, aminoacylated P-site fMet-tRNA_i_^Met^ were formed by programming 5 μM 70S ribosomes with 10 μM mRNA and 20 μM P- and A-site tRNAs. The LAR compound was also soaked into the pre-formed crystals of Tth 70S ribosomes complexed with *E. coli* protein Y (PY) essentially as described previously^55,56^. All complexes were formed in buffer containing 5 mM HEPES-KOH (pH 7.6), 50 mM KCl, 10 mM NH_4_Cl, and 10 mM Mg(CH_3_COO)_2_, and then crystallized in the buffer containing 100 mM Tris-HCl (pH 7.6), 2.9% (v/v) PEG-20K, 9–10% (v/v) MPD, 175 mM arginine, 0.5 mM β-mercaptoethanol. Crystals were grown by the vapor diffusion method in sitting drops at 19°C, stabilized and cryo-protected stepwise using a series of buffers with increasing MPD concentrations (25%, 30%, 35%) until reaching the final concentration of 40% (v/v) MPD as described previously^51–53,55,57^. Antibiotics LAR or LAR-B were included in all stabilization buffers (250 μM). After stabilization and cryo-protection, crystals were flash-frozen using a nitrogen cryo-stream at 80°K (Oxford Cryosystems, UK).

Collection and processing of the X-ray diffraction data, model building, and structure refinement were performed as described in our recent reports^53–57^. Diffraction data were collected at beamlines 24ID-C and 24ID-E at the Advanced Photon Source (Argonne National Laboratory). A complete dataset for each complex was collected using 0.979 Å irradiation at 100 K from multiple regions of the same crystal, using 0.3-degree oscillations. Raw data were integrated and scaled using XDS software (version from Jan 10, 2022)^58^. Molecular replacement was performed using PHASER from the CCP4 program suite (version 7.0)^59^. The search model was generated from the previously published structures of *T. thermophilus* 70S ribosome with bound mRNA and aminoacylated tRNAs (PDB entries 6XHW^55^ or 6XHV^55^. Initial molecular replacement solutions were refined by rigid-body refinement with the ribosome split into multiple domains, followed by positional and individual B-factor refinement using PHENIX software (version 1.17)^60^. Non-crystallographic symmetry restraints were applied to four parts of the 30S ribosomal subunit (head, body, spur, and helix 44) and four parts of the 50S subunit (body, L1-stalk, L10-stalk, and C-terminus of the L9 protein). Structural models were built in Coot (version 0.8.2)^61^. The statistics of data collection and refinement are compiled in **Supplementary Table 3**. All figures showing atomic models were rendered using PyMol software (www.pymol.org).

### Ex vivo blood infection model

*A. baumannii* C0286 was cultured overnight in MHB and diluted 1:200 in 0.5 ml human blood in 14 ml culture tubes (BioIVT, USA). Five microliters of compound or water were added, and the tubes were treated for 4 hours at 37°C, 220–250 rpm. The remaining cfu were enumerated by appropriate dilution in PBS and spotting on MHA plates. The experiment was conducted in three biological replicates.

### Animal studies

All animal studies were conducted according to guidelines set by the Canadian Council on Animal Care using protocols approved by the Animal Review Ethics Board at McMaster University under Animal Use Protocol #20-12-41. All animal studies were performed with 6–10-week-old female CD-1 mice (Harlan/Envigo, Cat. No. 030). Mice were rendered neutropenic by intraperitoneal administration of cyclophosphamide (Sigma-Aldrich, cat.#C0768–5G) at 150 mg kg^−1^ (body weight) four days before infection, followed by 100 mg kg^−1^ one day before infection. On the day of infection, mice were infected intramuscularly with 1 10^9^ cfu *A. baumannii* C0286per thigh, and their core body temperature and body weights were monitored. The treatment group was intraperitoneally administered lariocidin suspended in Ultrapure Distilled Water (Fisher Scientific, Cat. No.10-977-015) at 50 mg kg^−1^ at 1 h, 4 h, 8 h, and 20 h post-infection. The control group was intraperitoneally administered 10 μL/g (body weight) of Ultrapure distilled water. To quantify bacterial load, mice were sacrificed at 24 h or 48 h, or sooner if they reached the humane endpoint defined by our protocols. Spleen and thigh tissue were harvested and placed in Mixer Mill safeseal tubes containing 1mL of sterile PBS with beads and homogenized using a Mixer Mill at 30 Hz. Blood was collected in Lithium-Heparin Microtainer Tubes (VWR, Cat. No. CABD365965). Bacterial burden was enumerated by plating the tissue homogenates and blood on LB agar plates supplemented with ampicillin (200 μg/mL) and incubating overnight at 37 deg. C.

### Statistical methods

Unless stated otherwise in the legend, a two-tailed Mann–Whitney test was used to compare the two groups. A one-way ordinary ANOVA or two-way mixed-effects ANOVA with a Geiser-Greenhouse correction was used for multiple comparisons, as mentioned accordingly. Values were considered statistically significant at p < 0.05. Data were collected using Microsoft Excel, and statistical analyses were performed using Prism GraphPad v. 10.2.3.

## Figures and Tables

**Table 1:** Antimicrobial spectrum and mammalian toxicity of lariocidin

Organism	MIC (μg/ml)	
	MHB	RPMI-1640
*Bacillus subtilis* 168	1	n.d.
*Staphylococcus aureus* ATCC 29213	16–32	1
*S. aureus* USA300	64	0.5
*Escherichia coli* BW25113 WT	32	1
*E coli* BW25113 Δ*bamB*Δ*tolC*	2–4	≤0.25
*E coli* BW25113 Δ*bamB*	32	1
*E coli* BW25113 Δ*tolC*	2–4	0.5
*E coli* BW25113 *mcr-1*	64	n.d.
*E coli* C0617	>64	4
*E coli* C1507	64	2
*E coli* C1508	32	1
*Klebsiella pneumoniae* ATCC33495	>64	2
*K pneumoniae* C1557	>64	8
*K pneumoniae* C1559	>64	4
*Acinetobacter baumannii* ATCC17978	128	1
*A. baumannii* C0286	8	0.5
*A. baumannii* C1531	8	0.25–0.5
*A. baumannii* C0092	16–32	1–2
*A. baumannii* C0074	32	1
*Pseudomonas aeruginosa* PAO1	64–128	n.d.
*Mycobacterium tuberculosis* Ra	4–8	
*M. smegmatis* MC^2^ 155	1–2	
**Gut bacteria**		
*Lactobacillus plantarum* GC4	>256	
*Bifidobacterium breve* GC96	256	
*Clostridium perfringens* GC1584	>256	
*Blautia obeum* GC925	256	
*Bacteroides fragilis* GC73	>256	
**Cytotoxicity**		
HEK293 (IC_50_ μg/ml)	>1000	
Haemolysis (500 μg/ml)	<1%	
**Other**		
*Candida albicans* ATCC 90028	>256	

MIC – minimal inhibitory concentration; MHB – cation-adjusted Mueller-Hinton broth; n.d.=not determined; IC_50_= concentration inhibiting 50% of cell growth.

## Data Availability

Coordinates and structure factors were deposited in the RCSB Protein Data Bank with accession codes:
**9DFC** for the wild-type *T. thermophilus* 70S ribosome in complex with lariocidin-A, mRNA, aminoacylated A-site Phe-tRNA^Phe^, aminoacylated P-site fMet-tRNA_i_^Met^, and deacylated E-site tRNA^Phe^; **9DFD** for the wild-type *T. thermophilus* 70S ribosome in complex with lariocidin-B, mRNA, aminoacylated A-site Phe-tRNA^Phe^, aminoacylated P-site fMet-tRNA_i_^Met^, deacylated E-site tRNA^Phe^; **9DFE** for the wild-type *T. thermophilus* 70S ribosome in complex with lariocidin-A and protein Y; All previously published structures used in this work for structural comparisons were retrieved from the RCSB Protein Data Bank: PDB entries 6XHW, 6XHX, 6CAE, 4G5K, 4YBB, 4W2I. The complete genome sequence of *Paenibacillus* sp. M2 is available in NCBI GenBank with BioProject no. PRJNA1155614. The accession no. is under process.
